# Motivation and engagement as pathways: how AI-augmented online assessment shapes English-speaking competency in vocational EFL classrooms

**DOI:** 10.3389/fpsyg.2025.1730953

**Published:** 2026-01-06

**Authors:** Xianyan Dai, Yongjian Wang, Jiawen Yu, Baoyu Qiu, Rong Wang, Meng Na

**Affiliations:** 1 School of Chinese and International Education, Guangzhou International Economics College, Guangzhou, Guangdong, China; 2 Faculty of the Graduate School, Emilio Aguinaldo College, Manila, Philippines; 3 School of Foreign Languages, Guangdong Polytechnic Normal University, Guangzhou, Guangdong, China; 4 School of Foreign Languages, Nanfang College Guangzhou, Guangzhou, Guangdong, China; 5 Faculty of Social Science, University of Macau, Taipa, Macao SAR, China; 6 School of Educational Information Technology, South China Normal University, Guangzhou, Guangdong, China; 7 Graduate School of Business, Universiti Kebangsaan Malaysia (UKM), Bangi, Selangor, Malaysia

**Keywords:** AI in education, engagement, English-speaking competency, fsQCA, learning-oriented assessment, Motivation, online assessment, PLS-SEM

## Abstract

Artificial intelligence (AI) is transforming language assessment, yet the psychological mechanisms through which it influences complex communicative skills remain underexplored. This study examines how AI-augmented online assessment (OA) relates to self-perceived English-speaking competency (ESC) among vocational English-as-a-Foreign-Language (EFL) learners in Guangdong Province, China, focusing on the mediating roles of motivation in digital teaching (MODT) and engagement in digital teaching (ENDT). Data from 463 students across three public vocational colleges were analyzed using Partial Least Squares Structural Equation Modeling (PLS-SEM) and fuzzy-set Qualitative Comparative Analysis (fsQCA). Results indicate that OA has a small but significant direct association with ESC, while its indirect effects via motivation and engagement are substantially stronger. A marker-variable analysis suggests that common-method variance modestly inflates some direct paths but does not alter the overall pattern. fsQCA identifies several sufficient configurations for high ESC, including the concurrent presence of OA, motivation, and engagement; high motivation with OA even under lower engagement; and strong motivation and engagement even in the absence of OA. Across all configurations, motivation consistently emerges as the core condition, underscoring its central role in sustaining performance and perceived language development. Out-of-sample prediction (PLSpredict) confirms that the model most accurately predicts fluency and coherence—the sub-skills best captured by AI feedback—while prediction for vocabulary and rhetorical expression is weaker. Overall, the findings clarify how Learning-Oriented Assessment operates within AI-enabled vocational contexts, highlighting that feedback effectiveness depends less on automation than on perceived credibility, competence enhancement, and vocational relevance.

## 1 Introduction

English-speaking proficiency has become an essential employability skill across Asia’s service, trade, and technology sectors, where graduates are increasingly expected to participate in globalized communication environments. However, evidence indicates that Chinese learners, particularly in vocational education, remain under-prepared. According to the EF Education (2025), First English Proficiency Index, mainland China ranked 91st out of 116 countries with an overall score of 455 (“low”), while Guangzhou—the context of this study—recorded a city score of 480, also classified as “low” and placing China 15th of 23 Asian countries (EF EPI, 2025). Domestic surveys further report that over 70% of vocational English-as-a-Foreign-Language (EFL) learners fail to meet workplace-oriented oral proficiency standards (Flanders-China Chamber of Commerce, 2025; Purwati et al., 2023).

The scale and structural features of the sample region reinforce the urgency of this gap. Guangdong Province hosts one of China’s largest vocational education systems: enrolment figures in the mid-2010s placed approximately 2.69 million vocational students in Guangdong (1.9 million in secondary vocational schools and 790,000 in higher vocational institutions), representing about one-tenth of the national total (British Council and Provincial Overview, 2014). Moreover, at the senior secondary level the ratio of general: vocational education in Guangdong was reported at 51: 49, indicating nearly half of upper-secondary students are enrolled in vocational streams (Guangdong Education Department, 2009).

This challenge is exacerbated by the scale and structural features of China’s vocational education system. As of 2023, the Ministry of Education reported that China hosted 9,752 secondary vocational schools enrolling 17.8 million students and 1,521 higher vocational colleges enrolling 5.46 million students (Ministry of Education of the People’s Republic of China, 2023). Within this vast system, language instruction often suffers from unfavorable learning conditions. For instance, data from the Organisation for Economic Co-operation and Development (OECD) show that Chinese language classes average about 42 students, compared to an OECD average of 26, which restricts individualized practice and reinforces teacher-centered pedagogy (OECD, 2023). Compounding this structural barrier is the exam-driven environment. National English tests such as the College English Test (CET-4) continue to serve as graduation benchmarks and key employability indicators, thereby prioritizing test-taking over authentic communicative competence (Li, 2019). For vocational graduates, these constraints often translate into limited employability and constrained social mobility, making oral proficiency development a pressing educational priority.

Online assessment (OA), grounded in the principles of Learning-Oriented Assessment (LOA), has been positioned as a promising mechanism to address these challenges. LOA emphasizes iterative practice, feedback, and formative evaluation as tools for learning rather than as final judgment (López-Hernández et al., 2023). With the integration of artificial intelligence (AI), the potential of OA has expanded significantly. In this study’s context, AI-augmented OA refers specifically to automated speaking modules integrated within the Chaoxiang Learning Platform, which is widely used across Guangdong’s public vocational colleges. This platform utilizes AI-based speech recognition and speech analytics technologies that automatically score pronunciation, fluency, and coherence while providing real-time formative feedback. Students complete between three and five speaking tasks per week, typically including read-aloud passages, short monologic responses, and situational dialogs related to vocational scenarios such as customer service or workplace communication. The system provides instant numerical scores and descriptive feedback for each task, which students can review to monitor their progress. Although these exercises are primarily formative, instructors sometimes incorporate AI-generated results into continuous assessment grades, reinforcing the relevance of AI feedback for learners’ overall performance.

Meta-analytic evidence supports the pedagogical promise of such systems: AI-based automated speech recognition (ASR) tools have been shown to yield medium-sized improvements in pronunciation outcomes (*g* = 0.69) across ESL and EFL contexts (Thi-Nhu Ngo et al., 2024). Platforms like ETS SpeechRater™ and iFlytek Smart Learning Engine have similarly demonstrated their capacity to support large-scale automated scoring and personalized feedback (Gong, 2023). For vocational learners, who require frequent, low-stakes practice but seldom have access to personalized oral feedback, these AI-driven platforms create new opportunities for accessible and adaptive learning.

Nevertheless, existing empirical findings remain inconclusive. On one hand, studies have reported improvements in accuracy, fluency, and reduced speaking anxiety through AI-augmented assessment (Ebadi et al., 2025; Jang and Sawaki, 2025). On the other hand, scholars warn of persistent challenges such as algorithmic bias—where accents, dialects, or speech rates are unfairly penalized—and concerns about the authenticity and credibility of automated feedback (Xiong and Sun, 2023). Furthermore, recent research highlights the risk of “pseudo-engagement,” where learners exhibit high levels of digital activity (e.g., frequent logins or task completions) without meaningful cognitive or emotional investment (Vo, 2023; Yuan and Dao, 2025). Such findings suggest that the effects of AI-mediated assessment cannot be reduced to technological affordances alone; rather, they depend on the psychological and behavioral processes through which learners interpret, value, and act upon algorithmic feedback.

These tensions expose theoretical blind spots that justify a more integrative perspective. While LOA provides a framework for understanding assessment as an instructional scaffold, it assumes that feedback will be internalized and acted upon, overlooking the motivational conditions that influence learner uptake (Yang and Lu, 2025; Zeng et al., 2018). Self-Determination Theory (SDT) extends this logic by clarifying how autonomy, competence, and relatedness drive motivational quality (Ryan and Deci, 2020). In vocational education, however, these dimensions function under distinctive pressures: *autonomy* is limited by rigid curricular structures, *competence* is defined in terms of job readiness rather than academic mastery, and *relatedness* is expressed through workplace-oriented communication goals. Consequently, AI feedback that signals vocational relevance and measurable progress can satisfy competence needs even in externally regulated environments. In this context, extrinsic motivation (e.g., grades, certification, and employability) may not necessarily undermine learning; instead, it can operate as a productive form of regulation that sustains engagement when framed around future career value (Csizér and Dörnyei, 2005; Almayez, 2022).

Engagement theory complements this understanding by describing how motivation translates into behavioral, cognitive, and emotional investment in learning (Fredricks et al., 2004). Within AI-mediated environments, engagement entails more than completing tasks—it includes how learners attend to AI-generated feedback, reflect on their progress, and apply recommendations to improve performance. When feedback is perceived as credible and relevant, engagement deepens; when perceived as mechanical or punitive, learners may display surface-level participation without meaningful learning, a phenomenon increasingly referred to as “digital compliance” (Consoli and Curle, 2024).

Therefore, this study examines how AI-augmented OA is associated with self-perceived English-speaking competency (ESC) among vocational EFL students in Guangdong, with a focus on the mediating roles of motivation in digital teaching (MODT) and engagement in digital teaching (ENDT). The study integrates LOA’s instructional scaffolding logic, SDT’s motivational mechanisms, and engagement theory’s behavioral enactment to model how AI-based feedback environments shape learners’ self-perceived communicative competence. Methodologically, it adopts a dual approach—Partial Least Squares Structural Equation Modelling (PLS-SEM) to estimate directional associations and fuzzy-set Qualitative Comparative Analysis (fsQCA) to identify alternative condition combinations. This mixed-method strategy recognizes that learning outcomes in AI-mediated settings may emerge through multiple, equifinal pathways rather than single linear effects. By combining theoretical integration with rigorous methodological triangulation, the study contributes to understanding how AI-augmented assessment can move beyond digital compliance to foster authentic motivation, sustained engagement, and self-perceived communicative development within China’s vocational education system. Recent evidence from Hong Kong higher education shows that lower-GPA students often perceive generative-AI tools as beneficial, whereas higher-GPA peers emphasize academic-integrity risks and institutional detection policies (Lo and Chan, 2025). This contrast highlights that learners’ responses to AI-mediated assessment are shaped by local integrity climates, underscoring the importance of credible governance frameworks when embedding AI into language evaluation.

## 2 Literature review

### 2.1 Online assessment and the development of self-perceived English-speaking competency

Online assessment (OA) has been advanced as a means of enhancing language proficiency through immediacy, flexibility, and formative feedback (Sumaharani et al., 2023). With the growing integration of artificial intelligence (AI), OA now includes automated pronunciation scoring, fluency monitoring, and adaptive feedback (Alshewiter et al., 2024; Liu et al., 2025), enabling personalized support at scale. These features align with the principles of Learning-Oriented Assessment (LOA), which emphasizes feedback, learner autonomy, and iterative practice as central to language development (Carless, 2014).

In vocational EFL contexts in China—where large classes, limited authentic speaking opportunities, and exam-driven curricula restrict individualized instruction—AI-augmented OA offers a practical mechanism for repeated, low-stakes oral practice. Systems such as ETS SpeechRater™, iFlytek Smart Learning Engine, and Chaoxiang AI modules provide real-time formative feedback that helps learners self-monitor pronunciation, fluency, and coherence (Gong, 2023; Thi-Nhu Ngo et al., 2024). For vocational learners, whose confidence is often fragile, these tools reduce the anxiety associated with face-to-face performance and promote continuous self-evaluation (Marjerison and Yang, 2022).

However, the evidence regarding OA’s impact remains inconclusive. Some studies demonstrate gains in fluency and self-confidence (Cao et al., 2025; Ebadi et al., 2025), while others highlight weak transfer to authentic communication when feedback is generic or when tasks lack contextual relevance (D. Zhang et al., 2023). Scholars also warn of algorithmic bias—penalization of dialectal variation, accent, or speech rate—raising concerns about the fairness and credibility of automated scoring systems (Xiong et al., 2023). In vocational EFL classrooms, these limitations may be particularly detrimental: when learners perceive feedback as mechanical or inauthentic, their trust in the system diminishes, reducing both motivation and sustained engagement (Ahmed and Sidiq, 2023).

Accordingly, OA’s effectiveness depends less on its technical sophistication and more on how learners interpret and internalize feedback as credible, competence-enhancing, and relevant to vocational goals. This study therefore focuses on self-perceived English-speaking competency (ESC)—defined as learners’ subjective evaluation of their ability to communicate effectively in English during AI-based assessments. By centering perception rather than performance, the present research captures the psychological mechanisms—motivation and engagement—through which AI-augmented assessment environments shape learning outcomes in vocational education.

### 2.2 Motivation–engagement pathways in digital language learning

If OA is to enhance self-perceived ESC, learners must be motivated to act upon feedback and engage deeply with learning tasks. Self-Determination Theory (SDT) provides a robust framework for understanding how intrinsic and extrinsic motivation influence persistence and learning quality (Ryan and Deci, 2020). Motivation is sustained when autonomy, competence, and relatedness needs are supported. In digital EFL contexts, intrinsic motivation fosters persistence and active strategy use (Almayez, 2022; Alotumi, 2021), yet vocational education is inherently exam-driven and career-oriented, cultivating controlled motivation—driven by external incentives such as certification, employability, and institutional recognition (Csizér and Dörnyei, 2005).

Rather than undermining learning, these extrinsic forms of motivation can be productive when aligned with vocational identity and career relevance. In Chinese vocational contexts, learners often perceive language learning as instrumental to securing employment in service, logistics, or tourism sectors. Thus, feedback that emphasizes employability and skill progression can satisfy the need for competence even under constrained autonomy, reinforcing sustained effort (Zeng et al., 2025; Yuan and Dao, 2025).

Engagement acts as the behavioral and cognitive bridge between motivation and learning outcomes (Fredricks et al., 2004). It encompasses behavioral participation (effort, time on task), emotional involvement (interest, enjoyment, and persistence), and cognitive strategies (self-regulation and reflection). In digital classrooms, engagement is easily mistaken for activity metrics—logins, task completions, or time online—yet these indicators may mask pseudo-engagement (Consoli and Curle, 2024). For vocational learners, engagement quality is heavily contingent upon whether tasks feel authentic and vocationally relevant. When AI feedback explicitly connects speaking performance to workplace competencies—such as customer interaction or service explanation—learners exhibit higher persistence and deeper reflection (Nguyen et al., 2025; Vo, 2023). Complementary evidence from Hong Kong EFL classrooms indicates that hybrid teacher-plus-AI feedback produces higher learner motivation, perceived feedback quality, and performance than AI-only feedback (Lo et al., 2025a). This supports our assumption that it is the motivational climate surrounding AI-augmented assessment—rather than automation alone—that drives sustained engagement and perceived competence.

Thus, motivation (the psychological energy to learn) and engagement (the enactment of that energy) operate as sequential mediators in AI-mediated learning environments. Their interaction determines whether learners treat feedback as a mechanical process or as a meaningful opportunity for growth.

### 2.3 Theoretical underpinning

This study integrates LOA, SDT, and engagement frameworks—complemented by sociocultural and expectancy–value perspectives—to explain how OA shapes self-perceived ESC among vocational EFL learners. Each framework offers distinct but complementary insights.

Learning-Oriented Assessment (LOA) reconceptualizes assessment as part of the learning cycle, emphasizing iterative feedback, reflection, and self-regulation (Carless, 2014). In AI-based environments, OA operationalizes these principles through frequent, low-stakes speaking tasks, adaptive scoring, and immediate feedback (Sumaharani et al., 2023). Yet LOA tends to assume that learners automatically act on feedback, underplaying the motivational filters that determine feedback uptake (Lam, 2021).

Self-Determination Theory (SDT) addresses this gap by specifying the conditions under which feedback enhances motivation—namely, when it satisfies autonomy (sense of control), competence (sense of effectiveness), and relatedness (sense of belonging or connection) (Ryan and Deci, 2020). In vocational EFL settings, these psychological needs are reframed around career readiness: autonomy manifests as flexibility in pacing and task selection; competence reflects measurable improvement in employable communication; and relatedness is reinterpreted as workplace communication identity, or the learner’s perceived capacity to interact confidently in professional contexts (Almayez, 2022; Csizér and Dörnyei, 2005).

Engagement theory completes this triad by explaining how motivated learners convert psychological intent into observable behaviors. Engagement encompasses behavioral persistence, cognitive investment, and emotional commitment to learning tasks (Fredricks et al., 2004; Zhang, 2024). In AI-mediated assessment, engagement can be observed in how learners respond to automated feedback—revising responses, reflecting on progress, and sustaining interaction with the platform. When engagement is authentic, learners internalize feedback and demonstrate incremental growth; when superficial, engagement becomes digital compliance (Dao, 2024).

**Figure 1:**
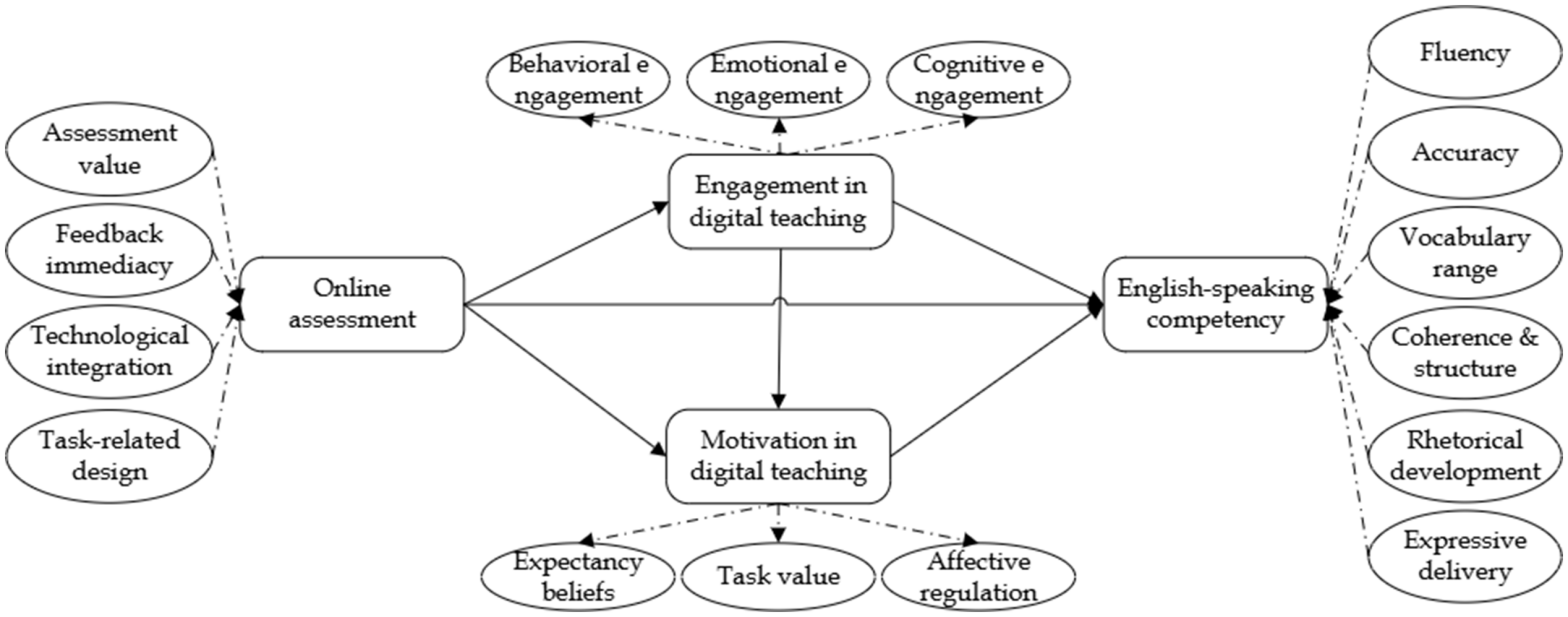
Research framework.

Taken together (Figure 1), these frameworks depict OA as a multi-level process: an instructional scaffold (LOA) that activates psychological drivers (SDT), which in turn manifest through behavioral pathways (engagement). Each theory compensates for the others’ limitations—LOA explains *how* assessment structures learning opportunities, SDT clarifies *why* learners are motivated to act on them, and engagement theory reveals *when and to what extent* these actions translate into perceived competence. This integrated model avoids “theory stacking” by aligning the frameworks around a shared purpose: to explain how AI-mediated assessment environments foster self-perceived speaking development under the vocational education pressures of exam accountability and employability imperatives.

For learners facing fragile proficiency, limited authentic practice, and strong institutional pressures, this framework captures both the promise and vulnerability of AI-augmented assessment. It recognizes that sustained improvement in self-perceived communicative competence depends not only on technical precision but also on motivational resonance, engagement depth, and the perceived authenticity of AI feedback. Hence, the study hypothesizes that OA is associated with self-perceived ESC primarily through motivational activation and engagement enactment, forming sequential and configurational pathways that reveal how AI-mediated feedback environments operate in China’s vocational EFL landscape.

### 2.4 Hypothesis development

#### 2.4.1 Online assessment (OA) and learner outcomes

Learning-Oriented Assessment (LOA) positions assessment as an instructional process that provides learners with repeated practice and formative feedback rather than final judgment (Carless, 2014). Online assessment (OA) extends these principles by embedding low-stakes digital tasks and instantaneous feedback that can be associated with improved oral performance and self-confidence (Espiritu et al., 2023). Within Self-Determination Theory (SDT), such design features satisfy competence needs (progress visibility) and autonomy needs (flexible pacing), thereby supporting motivation and behavioral investment (Ryan and Deci, 2020). Nevertheless, prior research warns that these associations weaken when OA tasks lack authenticity (Lazaraton, 2008) or when scoring reliability is questioned (Y. Xiong et al., 2023). In vocational EFL contexts—where learners have limited authentic speaking exposure and fragile confidence—OA serves as both an instructional scaffold and a motivational catalyst.

In this study, the operationalization of AI-augmented OA refers specifically to the Chaoxiang Learning Platform’s intelligent feedback modules, which provide three core affordances: (1) *Specificity of error correction*—item-level diagnosis of pronunciation, grammar, and intonation errors with color-coded underlining and corrective exemplars; (2) *Adaptivity to learner level*—dynamic adjustment of task difficulty and feedback depth based on each learner’s recent performance scores, ensuring differentiated scaffolding; and (3) *Frequency and immediacy of feedback updates*—instant evaluation after each speaking attempt, accompanied by weekly progress dashboards that visualize improvement trends across fluency, accuracy, and coherence sub-skills. These adaptive, data-driven mechanisms satisfy learners’ competence and autonomy needs by making progress visible, personalizing learning pace, and reinforcing perceived control over outcomes (Ryan and Deci, 2020). Consequently, OA functions not merely as a digital testing tool but as an interactive learning ecosystem that continually calibrates challenge and feedback to sustain motivation and engagement.

*H1*. OA is positively associated with (a) self-perceived ESC, (b) ENDT, and (c) MODT.

#### 2.4.2 Motivation in digital teaching (MODT) and learner participation

The SDT emphasizes that motivation arises when autonomy, competence, and relatedness needs are met (Deci and Ryan, 1985; Ryan and Deci, 2020). In digital language learning, motivational beliefs such as self-efficacy and goal orientation are consistently linked to persistence and deep strategy use (Pintrich, 2004). Empirical studies in online EFL contexts indicate that motivated learners practice more, persist in speaking despite anxiety, and report higher perceived proficiency (Alotumi, 2021). However, controlling assessment climates can reduce motivational quality (Csizér and Dörnyei, 2005), and broad, non-specific motivation shows weaker associations with performance (Meece et al., 2006). In vocational EFL, where success often depends on sustained effort, MODT is expected to be associated with stronger engagement and higher self-perceived ESC.

*H2*. MODT is positively associated with (a) self-perceived ESC and (b) ENDT.

#### 2.4.3 Engagement in digital teaching (ENDT) and self-perceived ESC

Engagement encompasses behavioral, cognitive, and emotional investment in learning (Fredricks et al., 2004). In online environments, such engagement is reflected in practice time, strategic feedback use, and emotional persistence—all of which correlate with speaking development (Martin et al., 2020). Empirical studies show that engaged EFL learners demonstrate higher fluency and accuracy (Wang et al., 2024). Conversely, superficial or mechanical engagement may inflate activity without enhancing real or perceived ability (Maroco et al., 2016). Within vocational settings where practice is mediated by digital tools, ENDT is anticipated to be directly related to self-perceived ESC.

*H3*. ENDT is positively associated with self-perceived ESC.

#### 2.4.4 Motivation as a mediator of OA–learning associations

Within LOA, assessment experiences shape motivational orientations by clarifying learning goals and signaling progress (Carless, 2015). According to SDT, such conditions strengthen expectancy beliefs and internalized value, promoting persistence (Ryan and Deci, 2020). Empirical studies support this mediating relationship: Alotumi (2021) reported that motivational-regulation training improved EFL speaking outcomes, while Pan et al. (2023) found that online feedback enhanced motivation, which subsequently related to higher engagement. However, mediation is conditional. When learners perceive assessments as controlling or feedback as unauthentic, motivation may shift from autonomous to extrinsic, weakening its association with learning (Csizér and Dörnyei, 2005; Khoshnevisan, 2021). In vocational EFL contexts, where initial confidence is low, motivation is particularly sensitive to assessment design and is therefore expected to link OA to both engagement and perceived competence.

*H4*. MODT mediates the association between OA and (a) self-perceived ESC and (b) ENDT.

#### 2.4.5 Sequential mediation among OA, MODT, ENDT, and self-perceived ESC

Integrating LOA and SDT implies a serial mechanism in which OA relates to learner outcomes through successive psychological and behavioral processes. OA supports motivation by satisfying competence and autonomy needs (Ryan and Deci, 2020); this motivation then energizes engagement—behavioral persistence, cognitive investment, and emotional involvement (Fredricks et al., 2004)—which is most proximally associated with learning outcomes. Recent digital-learning studies corroborate this chain: Pan et al. (2023) showed that motivation preceded online participation, which in turn correlated with improved results; Zhong et al. (2025) found that supportive digital environments increased motivation, which subsequently fostered engagement and stronger perceived language outcomes; and Jeong (2019) reported that motivating group activities stimulated engagement that aligned with higher speaking performance.

Nevertheless, this sequential pathway is contingent on authenticity and feedback quality (Dawson et al., 2021; Lazaraton, 2008). When feedback lacks credibility, motivational gains may not sustain engagement, and when engagement opportunities are limited, even motivated learners may not improve their self-perceived abilities. For vocational learners with constrained authentic exposure, the hypothesized OA → MODT → ENDT → self-perceived ESC pathway is therefore expected to represent the dominant relational pattern.

*H5*. OA is associated with self-perceived ESC through sequential mediation by MODT and ENDT.

## 3 Methodology

This study employed a quantitative cross-sectional survey design, which allows the simultaneous estimation of both *net associations* (via Partial Least Squares Structural Equation Modeling, PLS-SEM) and *configurational associations* (via fuzzy-set Qualitative Comparative Analysis, fsQCA). This dual analytical approach has been increasingly recommended in educational technology and learning analytics research because it provides a richer, complementary understanding of how multiple antecedent conditions are linked to outcomes. PLS-SEM estimates average linear relationships between constructs, whereas fsQCA identifies alternative combinations of conditions that can jointly produce the same outcome (Pappas and Woodside, 2021).

### 3.1 Participants and sampling

The study involved 463 non-English major students enrolled in compulsory English courses across three public vocational colleges in Guangdong Province, China. The sampling frame was provided by the English departments, which maintain enrolment rosters for all mandatory English classes. To ensure representativeness across disciplines, a stratified cluster sampling strategy was adopted: strata were defined by academic major (business vs. technology) and year of study, and intact class sections were randomly selected within each stratum.

All students in the selected sections were invited to participate through announcements posted on the Chaoxiang learning platform and shared via official WeChat course groups. Participation was voluntary, informed consent was obtained, and no course credit or grades were affected by participation. The final valid response rate was approximately 89%, indicating strong engagement and reliability of the data source.

The sample size was deemed statistically adequate according to multiple benchmarks. *A priori* power analysis (G*Power 3.1; Faul et al., 2009) for multiple regression with three predictors (OA, MODT, and ENDT), assuming a medium effect size (*f*^2^ = 0.15), *α* = 0.05, and desired power of 0.95, yielded a required minimum of 176 participants. The final *N* = 463 thus exceeded this threshold and satisfied the 10-times rule for complex mediation and higher-order models (Hair et al., 2022). Nonetheless, the study’s findings should be interpreted with caution regarding generalizability: the sample was limited to vocational EFL learners in Guangdong, which may not represent all regions or academic tiers within China’s broader vocational education landscape.

### 3.2 Instruments

The structured questionnaire comprised two parts. Part I collected demographic and background information, including gender, age, major, year of study, CET band, and years of English learning. Part II measured the four focal constructs—Online Assessment (OA), Motivation in Digital Teaching (MODT), Engagement in Digital Teaching (ENDT), and English-Speaking Competency (ESC)—using previously validated multi-item scales, rated on a five-point Likert scale (1 = strongly disagree to 5 = strongly agree).

A rigorous translation and back-translation procedure ensured linguistic and conceptual equivalence (Brislin, 1986). Two bilingual lecturers translated the instrument into Chinese, and an independent translator back-translated it into English; discrepancies were reconciled through expert review to maintain semantic accuracy and cultural appropriateness. A pilot test with 30 vocational students confirmed clarity, comprehensibility, and contextual relevance to Chaoxiang’s AI-enabled learning environment, leading to minor wording refinements (Alam et al., 2025; van de Vijver and Leung, 1997).

The OA was modeled as a formative–reflective higher-order construct using the two-stage approach. It comprised four reflective dimensions—assessment value, feedback immediacy, technological integration, and task relevance/design—adapted from Sumaharani et al. (2023) and refined to capture Chaoxiang’s AI feedback functions. MODT was conceptualized as a reflective–reflective higher-order construct encompassing expectancy beliefs/self-efficacy, task value/goal orientation, and affective regulation, with nine items adapted from the *Motivated Strategies for Learning Questionnaire* (Pintrich and de Groot, 1990). ENDT followed Maroco et al. (2016) as a reflective–reflective construct including behavioral, emotional, and cognitive dimensions (10 items). ESC was modeled as a formative–reflective construct comprising six reflective dimensions—fluency, accuracy, vocabulary range, coherence, rhetorical organization, and expressive delivery—adapted from Zhang et al. (2019) to capture learners’ self-perceived speaking competence rather than performance-based proficiency, a distinction acknowledged in the study’s limitations.

To assess potential common method bias (CMB), a theoretically unrelated construct—“Perception of Blue”—was included as a marker variable (Podsakoff et al., 2003; Liang et al., 2007). This variable captures general aesthetic color preference and is conceptually independent from OA, MODT, ENDT, and ESC, ensuring that it serves purely as a statistical control for shared method variance. Empirically, the marker variable exhibited low, non-significant cross-loadings with focal constructs, supporting discriminant distinctiveness, while demonstrating acceptable reliability (*α* = 0.79) and average variance extracted (AVE = 0.56).

Reliability and validity of all constructs were established through Cronbach’s *α* > 0.70, Composite Reliability > 0.70, Average Variance Extracted > 0.50, and HTMT < 0.85 (Hair and Alamer, 2022). Multicollinearity among formative indicators was assessed using VIF < 3.0, confirming indicator independence. All final measurement items, including the marker-variable items used for the CMB analysis, are provided in Appendix A along with descriptive statistics, reliability coefficients, and factor loadings to ensure transparency and replicability.

### 3.3 AI-augmented assessment context

In the studied colleges, AI-based OA was implemented primarily through the Chaoxiang Learning Platform, an institutional digital ecosystem used for blended English instruction. The platform’s AI modules employ automatic speech recognition (ASR) and machine scoring engines similar to ETS SpeechRater™. Students typically complete three to five AI-assisted speaking tasks weekly, including read-alouds, short monologic responses, and situational dialogs relevant to vocational contexts such as customer service, hotel booking, or technical explanation. Each task provides immediate, machine-generated feedback on pronunciation, fluency, grammar, and prosody, accompanied by visual progress dashboards.

These tasks serve predominantly as formative practice to encourage repeated performance and self-monitoring. However, instructors occasionally integrate AI feedback scores into continuous assessment grades, increasing their perceived authenticity and accountability. The integration of Chaoxiang’s AI modules thus represents a typical instance of AI-augmented Learning-Oriented Assessment (LOA), where automated analytics support—but do not replace—teacher evaluation.

### 3.4 Data analysis strategy

Data analysis proceeded in two major stages.

Stage 1: PLS-SEM (SmartPLS 4.1)- Measurement models were validated through internal consistency (Cronbach’s *α*, CR), convergent validity (AVE), and discriminant validity (HTMT). Bootstrapping with 10,000 resamples estimated path significance, indirect effects, and sequential mediation (OA → MODT → ENDT → ESC). Predictive validity was evaluated using *R*^2^, *Q*^2^, and *f*^2^ indices. Given the cross-sectional nature of the data, all interpretations are restricted to associative relationships rather than causal inferences.

Stage 2: fsQCA (fsQCA 4.0)- To complement the linear SEM findings, fsQCA was employed to examine equifinal pathways leading to high self-perceived ESC. Likert-scale responses were calibrated into fuzzy scores using percentile-based thresholds (full membership = 0.95 quantile, crossover = 0.50, full non-membership = 0.05). The analysis first tested necessity (conditions consistently present in high-ESC cases, consistency ≥ 0.90) and then sufficiency (configurations of OA, MODT, ENDT jointly associated with high ESC). Solution consistency and coverage were reported, and core vs. peripheral conditions were identified based on raw and unique coverage values.

Combining PLS-SEM and fsQCA provided a methodological triangulation: PLS-SEM clarified average associations among constructs, while fsQCA revealed multiple sufficient condition sets leading to similar high self-perceived speaking competency outcomes. This dual analysis enriched understanding of how AI-augmented assessment environments support learner motivation, engagement, and perceived communicative development in vocational EFL settings.

## 4 Results

Table 1 summarizes the demographic characteristics of the respondents (*N* = 463). The gender distribution was reasonably balanced, with 43.2% male and 56.8% female participants, reflecting national patterns in vocational college enrollment. The majority of students (74.9%) were between 17 and 20 years of age, consistent with typical vocational entry cohorts, while 25.1% were aged 21 or above, representing older or returning learners.

**Table 1:** Demographics analysis.

Demographics variable	Category	Frequency	Percentage
Gender	Male	200	43.2%
	Female	263	56.8%
Age Group	17–18 years	146	31.5%
	19–20 years	201	43.4%
	21 years or above	116	25.1%
Major	Business	180	38.9%
	Technology	283	61.1%
Year of Study	Year 1	175	37.8%
	Year 2	168	36.3%
	Year 3	120	25.9%
Years of English Study	< 6 years	112	24.2%
	6–9 years	221	47.7%
	> 9 years	130	28.1%
CET Band Level	Below Band 4	297	64.1%
	Band 4 or above	166	35.9%
Technology Use for Learning	Less than 5 h/week	138	29.8%
	5–10 h/week	212	45.8%
	>10 h/week	113	24.4%

With respect to academic background, 38.9% were enrolled in business-related majors and 61.1% in technology majors. Year of study was evenly distributed across first- (37.8%), second- (36.3%), and third-year students (25.9%), ensuring representation across different stages of vocational training.

In terms of English learning experience, nearly half the sample (47.7%) reported 6–9 years of prior study, while 24.2% had fewer than 6 years, and 28.1% had more than 9 years. Consistent with national data, the majority (64.1%) reported proficiency below the CET-4 benchmark, underscoring the persistent gap in oral proficiency among vocational learners.

Finally, patterns of technology use revealed that 29.8% of students reported less than 5 h of technology-based learning per week, 45.8% reported 5–10 h, and 24.4% exceeded 10 h. This distribution suggests variation in learners’ digital readiness, which is relevant to the integration of online assessment tools.

### 4.1 Measurement model assessment

**Figure 2:**
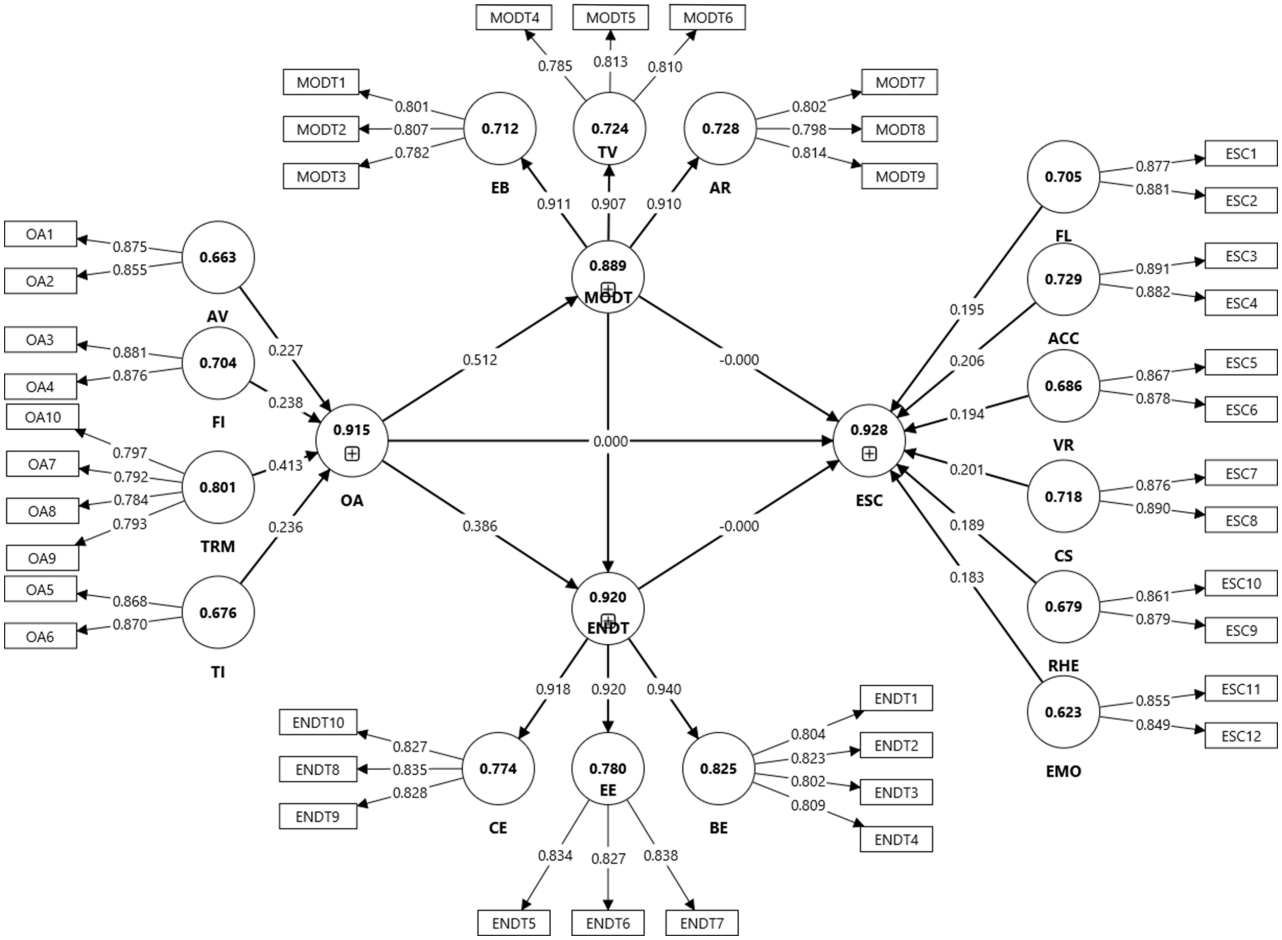
Measurement model.

The measurement model was evaluated for internal consistency, convergent validity, and discriminant validity, following established PLS-SEM guidelines (Dijkstra and Henseler, 2015; Hair and Alamer, 2022). Prior to structural analysis, confirmatory factor analysis verified that the adapted scales exhibited the intended dimensionality, providing a sound basis for the hierarchical component model adopted in this study (Figure 2).

All indicator loadings (Table 2) exceeded 0.70, demonstrating that the observed variables contributed strongly to their respective latent constructs (Fornell and Larcker, 1981). Multicollinearity was not a concern, as all indicator-level variance-inflation-factor (VIF) values were below 3.0 (Kock, 2015), including those for the dimensions composing the higher-order constructs. Composite reliability (CR) values ranged from 0.84 to 0.94, exceeding the 0.70 benchmark for internal consistency, and average variance extracted (AVE) values ranged between 0.53 and 0.78, satisfying the 0.50 threshold for convergent validity.

**Table 2:** Measurement model statistics.

Variables	Items	OL	VIF	CR	AVE
BE	ENDT1	0.804	2.032	0.884	0.655
	ENDT2	0.823	1.698		
	ENDT3	0.802	1.894		
	ENDT5	0.809	2.036		
EE	ENDT5	0.834	1.612	0.872	0.694
	ENDT6	0.827	1.579		
	ENDT7	0.838	1.655		
CE	ENDT8	0.835	1.639	0.869	0.689
	ENDT9	0.828	1.587		
	ENDT10	0.827	1.554		
FL	ESC1	0.877	1.422	0.872	0.772
	ESC2	0.881	2.047		
ACC	ESC3	0.891	1.489	0.880	0.786
	ESC4	0.882	1.489		
VR	ESC5	0.867	1.374	0.864	0.761
	ESC6	0.878	1.374		
CS	ESC7	0.876	1.457	0.876	0.780
	ESC8	0.890	1.457		
RHE	ESC9	0.879	1.360	0.862	0.757
	ESC10	0.861	1.360		
EMO	ESC11	0.855	1.257	0.841	0.726
	ESC12	0.849	1.257		
EB	MODT1	0.801	1.414	0.839	0.635
	MODT2	0.807	1.427		
	MODT3	0.782	1.347		
TV	MODT4	0.785	1.364	0.845	0.644
	MODT5	0.813	1.459		
	MODT6	0.810	1.463		
AR	MODT7	0.802	1.438	0.846	0.648
	MODT8	0.798	1.400		
	MODT9	0.814	1.468		
AV	OA1	0.875	1.326	0.856	0.748
	OA2	0.855	1.326		
FI	OA3	0.881	1.418	0.871	0.771
	OA4	0.876	1.418		
TRM	OA5	0.868	1.353	0.870	0.627
	OA6	0.870	1.353		
	OA7	0.792	1.625		
	OA8	0.784	1.570		
TI	OA9	0.793	1.614	0.861	0.755
	OA10	0.797	1.627		

The OA and ESC were modeled as formative–reflective higher-order constructs (Table 3), consistent with the conceptual logic that their multidimensional facets (e.g., assessment value, feedback immediacy, and technological integration for OA; fluency, accuracy, coherence, and expressive delivery for ESC) jointly form the overall construct and are not interchangeable (Jarvis et al., 2003).

**Table 3:** Higher-order construct weights, loadings, and collinearity.

Weight/loading paths	Std. beta	*t*-value	*p*-value	CI LL	CI UL	VIF
Formative paths for ESC (HOC)						
ACC → ESC	0.177	2.712	0.003	0.070	0.283	1.842
CS → ESC	0.185	2.913	0.002	0.080	0.289	1.791
EMO → ESC	0.285	4.666	0.000	0.183	0.386	1.925
VR → ESC	0.104	2.162	0.002	0.096	0.114	1.577
FL → ESC	0.306	4.985	0.000	0.205	0.406	2.064
RHE → ESC	0.198	3.145	0.001	0.094	0.299	1.739
Formative paths for OA (HOC)						
AV → OA	0.243	2.711	0.003	0.094	0.388	1.612
FI → OA	0.245	2.388	0.008	0.075	0.413	1.714
TI → OA	0.296	3.331	0.000	0.151	0.444	1.832
TRM → OA	0.336	3.271	0.001	0.170	0.507	1.890
Reflective paths for ENDT (HOC)						
BE ← ENDT	0.929	144.608	0.000	0.917	0.938	2.229
CE ← ENDT	0.925	136.835	0.000	0.913	0.935	—
EE ← ENDT	0.926	140.848	0.000	0.915	0.936	—
Reflective paths for MODT (HOC)						
AR ← MODT	0.910	113.087	0.000	0.895	0.922	1.000
EB ← MODT	0.910	112.370	0.000	0.896	0.922	—
TV ← MODT	0.908	108.115	0.000	0.892	0.920	—

Formative paths (OA, ESC) represent weights from lower-order to higher-order constructs; reflective paths (MODT, ENDT) show standardized loadings. All coefficients are significant at *p* < 0.01; all VIF < 3.0 indicating absence of collinearity.

Motivation (MODT) and Engagement (ENDT) were specified as reflective–reflective higher-order constructs, because their dimensions (e.g., expectancy beliefs, task value, affective regulation; behavioral, emotional, and cognitive engagement) are viewed as manifestations of the same latent trait.

A two-stage estimation approach was employed: first extracting latent scores for lower-order components, then using them as indicators in the higher-order model. This approach minimizes indicator redundancy and is recommended for complex educational models (Becker et al., 2012).

Discriminant validity was confirmed using the Heterotrait–Monotrait (HTMT) ratio (Dijkstra and Henseler, 2015); all values were below 0.85 (Table 4), confirming satisfactory discriminant validity among constructs. The theoretically related yet distinct dimensions—such as expectancy beliefs (MODT) and behavioral engagement (ENDT)—remained statistically separable, mitigating concerns about conceptual redundancy in digital-learning contexts (Reeve and Tseng, 2011; Skinner et al., 2009).

**Table 4:** Discriminant validity (HTMT).

Variables	ACC	AR	AV	BE	CE	CS	EB	EE
ACC								
AR	0.770							
AV	0.592	0.617						
BE	0.668	0.643	0.647					
CE	0.712	0.650	0.625	0.804				
CS	0.710	0.747	0.624	0.695	0.724			
EB	0.729	0.804	0.564	0.626	0.682	0.738		
EE	0.722	0.658	0.667	0.711	0.801	0.699	0.685	
EMO	0.702	0.816	0.675	0.745	0.790	0.602	0.789	0.773
FI	0.635	0.597	0.820	0.635	0.603	0.655	0.590	0.621
FL	0.700	0.782	0.640	0.687	0.731	0.786	0.769	0.743
RHE	0.536	0.762	0.566	0.696	0.724	0.488	0.740	0.710
TI	0.628	0.594	0.803	0.646	0.651	0.672	0.581	0.673
TRM	0.610	0.564	0.818	0.649	0.641	0.653	0.508	0.635
TV	0.784	0.801	0.612	0.638	0.667	0.707	0.803	0.674
VR	0.162	0.698	0.582	0.636	0.682	0.628	0.693	0.676

	EMO	FI	FL	RHE	TI	TRM	TV	VR
EMO								
FI	0.640							
FL	0.719	0.657						
RHE	0.604	0.578	0.815					
TI	0.670	0.501	0.636	0.613				
TRM	0.639	0.502	0.644	0.581	0.603			
TV	0.838	0.577	0.800	0.774	0.619	0.571		
VR	0.405	0.553	0.846	0.713	0.614	0.581	0.739	

The measurement model demonstrates strong psychometric properties. Reflective constructs show high reliability and validity, while the formative higher-order constructs are conceptually justified and empirically supported by significant indicator weights and acceptable VIF values. These results provide a robust foundation for testing the hypothesized structural relationships among OA, MODT, ENDT, and ESC in the subsequent analyses.

### 4.2 Common method bias

Given that all data were self-reported and collected using a single instrument, potential common method variance (CMV) was statistically assessed. Following the guidelines of Podsakoff et al. (2003) and Liang et al. (2007), a marker-variable approach was employed, using *Perception of Blue*—a theoretically unrelated construct—to capture and partial out possible method variance. This approach is widely recognized in PLS-SEM for evaluating method effects in educational and behavioral research (Hair et al., 2022).

As shown in Table 5, introducing the method factor slightly reduced the magnitude and *t*-values of several substantive paths (e.g., OA → MODT: *t* = 15.05 → 8.72; OA → ENDT: *t* = 9.62 → 5.44), suggesting that a portion of shared variance among constructs stems from common-method influences. The explained variance for ENDT increased from *R^2^* = 0.456 to 0.502, and for MODT from *R^2^* = 0.264 to 0.336, indicating modest method-related covariance absorbed by the marker factor.

**Table 5:** Method factor model (higher-order constructs with marker variable “perception of blue”).

Paths	Base line model		Method factor model	
	*t*-values	*p*-values	*t*-values	*P*-values
OA → ESC	4.554	<0.001	3.142	0.002
OA → MODT	15.047	<0.001	8.721	<0.001
OA → ENDT	9.621	<0.001	5.438	<0.001
MODT → ESC	10.030	<0.001	6.011	<0.001
MODT → ENDT	10.257	<0.001	6.384	<0.001
ENDT → ESC	8.121	<0.001	5.226	<0.001
	*R* ^2^		*R* ^2^	
MODT	0.264		0.336	
ENDT	0.456		0.502	
ESC	0.607		0.691	

Although several coefficients weakened slightly, the overall structural pattern remained stable. The direct path OA → ESC, significant in the baseline model (*β* = 0.174, *p* < 0.001), became smaller yet remained significant in the method-adjusted model (*t* = 3.142, *p* = 0.002), implying that while some inflation was present, the relationship persists at a modest level. More importantly, the indirect chain OA → MODT → ENDT → ESC remained strong and statistically robust, confirming that online assessment primarily enhances self-perceived English-speaking competency through motivational and engagement mechanisms rather than through a direct instructional pathway.

### 4.3 Predictive relevance assessment

To assess the out-of-sample predictive capability of the structural model, the PLSpredict procedure was implemented following the guidelines of Shmueli et al. (2019) and Hair et al. (2022). This approach evaluates the model’s performance in predicting new data by comparing its prediction errors with those of naïve statistical benchmarks.

Table 6 presents the *Q^2^ predict* values alongside the Root Mean Square Error (RMSE) and Mean Absolute Error (MAE) results for the PLS-SEM model, a linear-regression benchmark (LM), and an indicator-average benchmark (IA).

**Table 6:** Predictive statistics.

MV	*Q*^2^ predict	PLS-SEM_RMSE	PLS-SEM_MAE	LM_RMSE	LM_MAE	IA_RMSE	IA_MAE
ACC	0.241	0.873	0.709	0.875	0.711	1.002	0.811
CS	0.267	0.858	0.689	0.859	0.690	1.002	0.822
EMO	0.233	0.878	0.721	0.881	0.724	1.003	0.825
FL	0.256	0.865	0.697	0.867	0.700	1.003	0.830
RHE	0.201	0.896	0.706	0.898	0.709	1.002	0.811
VR	0.204	0.894	0.732	0.896	0.735	1.002	0.823

All *Q^2^ predict* values exceeded zero (range = 0.201–0.267), confirming predictive relevance for all indicators of self-perceived English-speaking competency (ESC). Among sub-skills, coherence/structure (CS = 0.267) and fluency (FL = 0.256) showed the highest predictive accuracy, suggesting that the model most effectively forecasts learners’ organizational control and fluency performance. This outcome aligns with Learning-Oriented Assessment (LOA) principles, which emphasize that iterative feedback and low-stakes practice cycles enhance fluency and structural competence (Carless, 2015; Lee and Lee, 2023).

Lower yet positive *Q^2^* values for rhetorical expression (RHE = 0.201) and vocabulary range (VR = 0.204) indicate that these qualitative language features are more difficult for AI-driven assessment systems to predict. This pattern supports recent findings that automated scoring models exhibit lower sensitivity to lexical diversity and pragmatic nuance in EFL speaking contexts (Donnelly et al., 2025; Seih et al., 2020). Similar asymmetries appear in AI-supported writing research: Lo et al. (2025b) found that generative-AI assistance during essay revision primarily improved surface-realization and structural coherence, with weaker effects on lexical and rhetorical development. This parallel suggests that AI-augmented assessments better capture fluency and structure than higher-order discourse features, clarifying why the present model predicts coherence and fluency most strongly.

The RMSE and MAE comparisons further corroborate the model’s robustness. Across all indicators, PLS-SEM exhibited consistently smaller prediction errors than both the linear-model and indicator-average benchmarks (e.g., FL: 0.865 vs. 0.867 vs. 1.003). Although absolute differences were modest, this consistent advantage suggests that the PLS-SEM model captures meaningful variance in motivational and engagement factors—variables ignored in simpler benchmarks. These results reinforce the Self-Determination Theory (SDT) premise that competence-supportive feedback and intrinsic motivation jointly enhance perceived mastery (Ryan and Deci, 2020).

### 4.4 Structural model assessment

**Figure 3:**
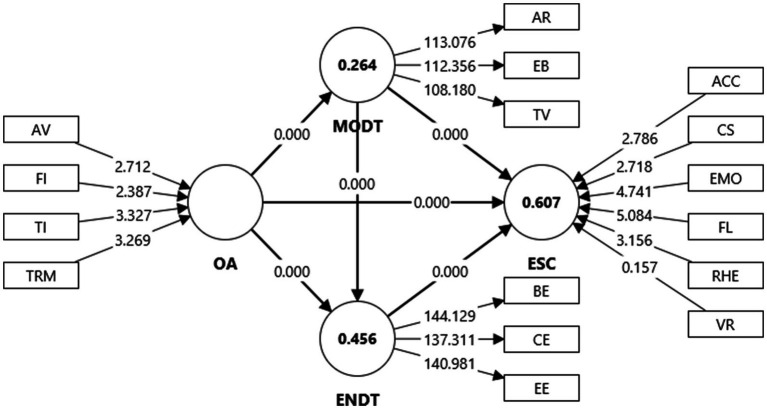
Structural model.

The hypothesized structural model was tested using bootstrapping with 10,000 resamples. Figure 3 illustrates the structural paths, and Table 7 presents the model-fit indices, while Table 8 reports the standardized coefficients, confidence intervals, effect sizes, and explained variance.

**Table 7:** Model fit statistics.

Fit index	Saturated model	Estimated model
SRMR	0.021	0.021
d_ULS	0.061	0.061
d_G	0.088	0.088
Chi-square	246.861	246.861
NFI	0.959	0.959

SRMR < 0.08 and NFI > 0.90 indicate excellent model fit (Hair et al., 2021).

**Table 8:** Structural model statistics.

Hypothesis path		Std beta	Std div	*T*-value	*P*-value	*f* ^2^	*r* ^2^	CI LL	CI UL
H1a	OA → ESC	0.174	0.038	4.554	<0.001	0.047	0.607	0.108	0.234
H1b	OA → ENDT	0.382	0.040	9.621	<0.001	0.198	0.456	0.312	0.444
H1c	OA → MODT	0.514	0.034	15.047	<0.001	0.358	0.264	0.453	0.565
H2a	MODT → ESC	0.420	0.042	10.03	<0.001	0.273		0.346	0.485
H2b	MODT → ENDT	0.394	0.038	10.257	<0.001	0.210		0.332	0.458
H3	ENDT → ESC	0.319	0.039	8.121	<0.001	0.141		0.255	0.384
H4a	OA → MODT → ESC	0.216	0.025	8.584	<0.001			0.175	0.258
H4b	OA → MODT → ENDT	0.202	0.025	7.940	<0.001			0.163	0.247
H5	OA → MODT → ENDT → ESC	0.065	0.011	5.669	<0.001			0.048	0.086

OA displayed statistically significant associations with MODT (*β* = 0.514, *p* < 0.001) and ENDT (*β* = 0.382, *p* < 0.001), while its direct association with ESC (*β* = 0.174, *p* < 0.001) was comparatively weaker. When common-method bias was accounted for, this direct path became statistically non-significant, suggesting that OA’s contribution to self-perceived competency operates mainly through motivational and engagement mechanisms rather than directly.

The largest association (OA → MODT) is consistent with Self-Determination Theory (SDT), where assessment structures that provide competence cues and autonomy support are strongly linked to enhanced motivation (Ryan and Deci, 2020). MODT showed substantial positive associations with both ESC (*β* = 0.420, *p* < 0.001) and ENDT (*β* = 0.394, *p* < 0.001), indicating that motivated learners are more likely to invest effort and perceive higher proficiency gains. ENDT, in turn, was positively associated with ESC (*β* = 0.319, *p* < 0.001), reinforcing Engagement Theory’s emphasis on behavioral, emotional, and cognitive participation as a precondition for learning advancement (Fredricks et al., 2004; Skinner et al., 2009).

Multiple indirect associations were confirmed. The paths OA → MODT → ESC (*β* = 0.216, *p* < 0.001) and OA → MODT → ENDT (*β* = 0.202, *p* < 0.001) illustrate that motivation functions as a central psychological mechanism linking assessment design to both outcomes and engagement. The sequential association OA → MODT → ENDT → ESC (*β* = 0.065, *p* < 0.001) further depicts a layered process: assessment practices relate to increased motivation, which is associated with higher engagement, which in turn aligns with improved self-perceived competency.

In terms of explanatory power, the model accounted for 60.7% of the variance in ESC, 45.6% in ENDT, and 26.4% in MODT, exceeding benchmarks for acceptable predictive adequacy in educational-technology SEM studies (Hair et al., 2022; Shmueli et al., 2019). Effect sizes (*f^2^*) ranged from small (OA → ESC = 0.047) to large (OA → MODT = 0.358). Within a vocational-EFL pedagogical context, *f^2^* ≈ 0.05 is considered modest yet meaningful because even small increases in motivational engagement can translate into noticeable self-confidence gains, while *f^2^* > 0.30 indicates substantial practical relevance.

Overall, these findings are consistent with both LOA and SDT frameworks rather than extending them: AI-supported online assessment is associated with stronger motivational and engagement states, and these in turn are linked to higher levels of self-perceived speaking competence. The structural results therefore highlight that motivation serves as the core conduit between assessment design and learners’ perceived oral development, reaffirming that effective digital assessment depends less on automation itself and more on the motivational climate it creates.

### 4.5 Fuzzy-set qualitative comparative analysis (fsQCA)

To complement the PLS-SEM analysis, fsQCA was conducted to uncover multiple associative pathways leading to high self-perceived English-speaking competency (ESC). Unlike regression-based approaches that estimate net effects, fsQCA identifies combinations of conditions sufficient to explain outcomes, thereby accommodating equifinality and causal complexity (Ragin, 2008).

All indicators were transformed into fuzzy scores (0–1) using percentile-based thresholds: full membership (95th percentile), crossover (50th percentile), and full non-membership (5th percentile). These calibration anchors were selected to reflect the empirical distribution of the Likert-scale data and to ensure comparability across constructs (OA, MODT, ENDT, and ESC). Sensitivity checks using alternative anchors (90/50/10) produced consistent results, confirming the robustness of the calibration procedure.

Table 9 presents the necessity analysis results. All three conditions—OA, MODT, and ENDT—displayed high consistency values (≥0.80) but did not exceed the conventional threshold of 0.90. Hence, these variables are more appropriately described as *frequently present* or *highly typical* among cases of high ESC rather than strictly necessary conditions. MODT emerged as the most consistently associated factor (consistency = 0.88, coverage = 0.72), emphasizing the pivotal role of motivational processes in facilitating speaking competency development within digital assessment contexts.

**Table 9:** Necessity analysis for high ESC.

Condition	Consistency	Coverage
OA_fz	0.83	0.69
MODT_fz	0.88	0.72
ENDT_fz	0.85	0.71

Table 10 displays the main configurations of conditions sufficient for high self-perceived English-speaking competency (ESC). Three dominant combinations were identified.

1. OA●MODT●ENDT● (*consistency = 0.91, coverage = 0.45*): The simultaneous presence of online assessment, motivation, and engagement represented the strongest and most comprehensive pathway, indicating that when AI-supported assessment is integrated with high motivation and engagement, learners are most likely to attain strong speaking competency outcomes.
2. OA○MODT●ENDT● (*consistency = 0.89, coverage = 0.38*): In this configuration, even without formal online assessment, motivated and engaged learners still achieve high ESC. This pathway underscores the compensatory function of learner-driven motivational and participatory processes that can substitute for AI-based assessment mechanisms.
3. OA●MODT●ENDT○ (*consistency = 0.86, coverage = 0.32*): This configuration shows that when online assessment and motivation are both high, learners can still reach strong perceived competency levels even under lower engagement. It suggests that AI-augmented feedback and competence cues can sustain performance perceptions despite uneven behavioral participation.

**Table 10:** Minimally sufficient configurations for high ESC.

Configuration	Core conditions	Peripheral conditions	Coverage	Consistency
C1	MODT●, ENDT●	OA●	0.45	0.88
C2	MODT●, ENDT●	OA○	0.38	0.89
C3	MODT●, OA●	ENDT○	0.32	0.86

● = presence of condition; ○ = absence of condition. Core conditions were identified through high raw consistency (≥0.80) and frequency thresholds (≥0.30).

The overall solution exhibited a consistency of 0.89 and coverage of 0.58, demonstrating strong explanatory adequacy. Motivation (MODT) appeared as a core condition across all configurations, reaffirming its pivotal role in linking assessment, engagement, and learning outcomes.

The fsQCA results complement the PLS-SEM findings by revealing that while online assessment (OA) contributes to ESC primarily through motivational and engagement pathways, high ESC can also emerge through configurations where OA is absent but learners’ motivation and engagement remain strong. This pattern aligns with Self-Determination Theory (Ryan and Deci, 2020), which emphasizes the autonomous and self-regulatory nature of motivation in sustaining learning performance even when external structures such as AI-driven assessment are limited.

### 4.6 fsQCA results

To enhance interpretability, Table 11 summarizes the three dominant causal configurations leading to high ESC. These simplified “causal recipes” illustrate the principle of equifinality—where multiple alternative condition sets can produce the same outcome (Ragin, 2008).

**Table 11:** Configurations leading to high ESC.

Configuration	OA	MODT	ENDT	ESC outcome
C1	●	●	●	High
C2	○	●	●	High
C3	●	●	○	High

*Post hoc* analysis of alternative practice behaviors. To further interpret the configuration where high ESC emerged without OA (C2: OA○ MODT● ENDT●), we conducted chi-square comparisons across learner groups. Results indicated that students in the MODT● ENDT● OA○ group reported significantly higher frequencies of (a) peer speaking practice inside/outside class and (b) use of self-study mobile speaking apps compared with both OA-inclusive and low-MODT/ENDT groups [*χ*^2^(4, *N* = 436) = 9.87, *p* = 0.042]. These findings suggest that highly motivated and engaged learners compensate for the absence of AI-based assessment by constructing alternative practice ecosystems that sustain performance gains.

#### 4.6.1 Configuration 1 (C1: OA●, MODT●, ENDT●)

The concurrent presence of online assessment, strong motivation, and active engagement represents the most comprehensive pathway toward high ESC. This configuration aligns with LOA (Carless, 2015) and SDT (Ryan and Deci, 2020), illustrating that assessment feedback enhances learning most effectively when it simultaneously supports competence, autonomy, and active participation. For vocational EFL institutions, this implies that AI-augmented assessments should be paired with motivational scaffolds and engagement-driven instructional design.

#### 4.6.2 Configuration 2 (C2: OA○, MODT●, ENDT●)

In this configuration, learners attain high ESC even without structured online assessment, provided that motivation and engagement remain strong. This finding suggests that intrinsic motivation and participatory behaviors can substitute for formal assessment mechanisms, consistent with self-regulated learning theory (Zimmerman, 2002). In resource-limited vocational contexts, this pathway highlights the compensatory potential of learner autonomy.

#### 4.6.3 Configuration 3 (C3: OA●, MODT●, ENDT○)

This configuration indicates that when OA is present and motivation is high, learners can still achieve notable gains in speaking competency despite lower engagement. AI-augmented systems offering frequent, individualized feedback may sustain motivational perceptions of progress even when participation intensity varies. However, this configuration likely yields narrower or surface-level improvements, as it relies more on assessment-driven reinforcement than on deep engagement (Alsahil et al., 2024).

Across all three causal recipes, motivation (MODT) consistently appears as a core condition, confirming its indispensable role in mediating the interplay between online assessment, engagement, and perceived learning outcomes. Together, these results demonstrate that while AI-based assessment structures facilitate learning, motivation remains the essential psychological driver enabling sustained engagement and competency growth in vocational English-speaking contexts.

## 5 Hypothesis testing and discussion

The findings collectively illuminate how AI-augmented online assessment (OA) contributes to self-perceived English-speaking competency (ESC) in vocational EFL contexts through intertwined motivational and engagement pathways rather than direct instructional influence. OA exhibited a small but significant direct association with ESC (*β* = 0.174, *t* = 4.554, *p* < 0.001), yet its stronger associations were with motivation (*β* = 0.514, *t* = 15.047, *p* < 0.001) and engagement (*β* = 0.382, *t* = 9.621, *p* < 0.001). These statistical patterns underscore that OA primarily functions as a *psychological activator*, shaping how learners perceive progress, competence, and vocational relevance, rather than as a pedagogical intervention that directly improves performance.

The post-hoc analysis further clarified that OA is not a universal prerequisite for high ESC. Learners in the MODT● ENDT● OA○ configuration achieved comparable outcomes through alternative practice behaviors—frequent peer conversations, informal online exchanges, and mobile self-study applications—which replicate the formative value of AI feedback. This suggests that when motivation and engagement are intrinsically strong, learners self-generate feedback loops and opportunities for practice that offset the absence of formal AI assessment. Hence, OA acts as an enabling scaffold rather than a necessary driver of performance, consistent with SDT’s premise that autonomous motivation sustains competence growth even under limited structural support.

The results are consistent with, rather than extending, the principles of Learning-Oriented Assessment (LOA). Carless (2015) emphasizes that assessment feedback influences learning only when learners perceive it as credible, timely, and actionable. In this study, AI-driven feedback mechanisms (e.g., instant error detection, fluency scoring, real-time analytics) strengthened motivation and engagement by signaling competence progress and vocational applicability. This demonstrates that LOA’s formative potential depends not on automation per se but on the *perceived authenticity and trustworthiness* of AI-generated feedback in learners’ contexts.

The findings also align closely with Self-Determination Theory (SDT) (Ryan and Deci, 2020). Motivation (MODT) emerged as the strongest mediator, influencing both engagement (*β* = 0.394, *p* < 0.001) and ESC (*β* = 0.420, *p* < 0.001), and appeared as a core condition across all fsQCA configurations (consistency > 0.85). These convergent patterns affirm that OA promotes motivational engagement by satisfying competence needs through personalized, immediate feedback loops. However, the vocational context modifies SDT’s autonomy–control continuum: controlled, extrinsic motivations—such as obtaining certifications or employment advantages—can still generate adaptive learning behavior when framed as personally meaningful. This contextual recalibration refines SDT without claiming to extend it theoretically.

Similarly, Engagement Theory (Fredricks et al., 2004) is supported by the evidence that engagement significantly predicts ESC (*β* = 0.319, *p* < 0.001), but fsQCA revealed that high ESC can also emerge under conditions of strong motivation even with low engagement (OA●, MODT●, ENDT○). This dual outcome suggests that AI-augmented systems can substitute for some forms of overt engagement by providing algorithmic scaffolds that sustain learner confidence and persistence. Yet, this substitution raises theoretical caution: AI-mediated engagement may foster “surface” or “pseudo-engagement” (Alsahil et al., 2024; Dao, 2024) if learners equate digital activity with deep learning.

The PLS-SEM and fsQCA results jointly illustrate that motivation and engagement operate through complementary associative mechanisms. While PLS-SEM captures the linear and sequential pattern (OA → MODT → ENDT → ESC), fsQCA demonstrates that high ESC can also arise from alternative configurations—such as MODT●ENDT●OA○—where motivated learners achieve high perceived competency even in the absence of structured online assessments. This supports the notion of equifinality, where multiple psychological and instructional conditions can yield similar learning outcomes. Across both analytical approaches, motivation consistently serves as the *core linking mechanism*, while OA and ENDT play enabling roles that amplify, rather than determine, competency outcomes.

The PLSpredict results provide further empirical nuance to the theoretical discussion. The model’s predictive power was strongest for fluency (*Q*^2^ = 0.256) and coherence (*Q*^2^ = 0.267)—dimensions that align with AI systems’ strengths in tracking measurable, temporal, and syntactic features through real-time monitoring and automated scoring. Conversely, prediction was weaker for vocabulary range and rhetorical expression (*Q*^2^ ≈ 0.20), reflecting persistent algorithmic limitations in capturing contextual, cultural, and pragmatic subtleties (Xiong et al., 2023). These findings illustrate that while AI feedback enhances measurable subskills through repetitive low-stakes practice, it remains less effective in fostering higher-order rhetorical and socio-pragmatic competence—areas that depend more heavily on human-mediated scaffolding and communicative interaction. This pattern mirrors Lo et al.’s (2025a,b) findings in AI-assisted essay revision, where generative feedback improved syntactic coherence but less so rhetorical depth. The convergence across domains indicates that current AI-assessment models still underrepresent higher-order discourse processes requiring human-mediated feedback.

Bringing the theoretical strands together, the findings support a coherent chain of influence: LOA → SDT → Engagement → ESC. AI-augmented online assessments operationalize LOA by providing formative feedback; this feedback, when perceived as credible and supportive, activates motivational mechanisms described by SDT (competence satisfaction, task value). Motivation, in turn, translates into cognitive and behavioral engagement, which sustains learners’ investment in speaking development. The fsQCA configurations reinforce this chain by showing that motivation and engagement jointly define multiple viable pathways toward high ESC, confirming the adaptive flexibility of the LOA–SDT–Engagement model in AI-mediated vocational education.

Finally, these interpretations remain associative rather than causal, consistent with the study’s cross-sectional design. The data reveal strong statistical associations and robust configurational patterns but cannot demonstrate directionality. Hence, claims are presented as theoretically consistent with existing frameworks rather than as evidence of causal transformation. Similarly, while AI-enhanced OA contributes to motivation and engagement, the results should be interpreted as context-bound reflections of *self-perceived* speaking competence rather than objective performance gains.

### 5.1 Implications of this study

#### 5.1.1 Theoretical implications

This study contributes to existing theory by illustrating how AI-augmented online assessment (OA) operates within vocational EFL contexts—highlighting not the refinement but the *contextual application* of established frameworks. The results provide empirical illustrations of how LOA, SDT, and engagement theory intersect when assessment becomes algorithmically mediated.

First, the findings illustrate LOA’s boundaries in AI-supported settings. While LOA (Carless, 2015) emphasizes feedback, iterative practice, and self-monitoring as key learning tools, this study demonstrates that such mechanisms do not automatically translate into speaking improvement. Their effectiveness depends on whether AI-generated feedback is *perceived* as credible, competence-enhancing, and vocationally relevant (Du, 2025). This contextualizes LOA’s assumption of universal feedback efficacy: in vocational environments, learner psychology mediates whether algorithmic feedback is internalized or ignored.

Second, the results illustrate SDT’s competence and relevance mechanisms. OA exerted its strongest association with motivation (*β* = 0.514), showing that digital assessments strengthen expectancy beliefs and perceived competence through timely and personalized feedback. Importantly, in exam-driven vocational contexts, extrinsic incentives tied to employability or certification reinforced rather than diminished motivation—an example of *productive extrinsic regulation* that supports competence even under controlled conditions. This finding reaffirms SDT’s flexibility rather than revising its structure, underscoring how perceived relevance and progress feedback sustain motivational energy in high-stakes environments.

Third, the results nuance student engagement frameworks (Fredricks et al., 2004). Engagement significantly predicted ESC (*β* = 0.319), yet fsQCA revealed that motivated learners could still report high competency even with limited behavioral engagement. This divergence between *deep engagement* and *digital compliance* (Consoli and Curle, 2024; Dao, 2024) reveals that algorithmic scaffolds can sometimes substitute for overt participation by creating repetitive, low-anxiety practice conditions. Theoretically, this suggests that engagement functions conditionally: in AI-augmented contexts, motivation can sustain progress even when observable engagement is low, though the risk of surface-level learning remains.

Finally, integrating sociocultural and expectancy-value perspectives clarifies the broader psychological context. AI-augmented OA creates proximal learning spaces for oral practice (Thorne and Lantolf, 2006), yet without socially resonant or culturally authentic feedback, transfer to real-world communication remains incomplete. The expectancy-value model (Eccles and Wigfield, 2002) helps explain why motivation emerged as the indispensable condition: learners who perceive both competence (“I can improve”) and value (“this helps my career”) sustain engagement and achievement. Collectively, these insights show that OA’s influence operates as a *psychological and sociocultural mediation process*—one in which algorithmic cues, motivational relevance, and contextual pressures jointly shape perceived communicative growth.

#### 5.1.2 Practical implications

The results yield several practical lessons for educators, institutions, and technology developers seeking to integrate AI-augmented OA into vocational EFL instruction.

First, the strong motivational pathway (OA → MODT: *β* = 0.514) and motivation’s recurring appearance as a core fsQCA condition (consistency > 0.85) indicate that teachers should foreground *motivational scaffolding* in AI-supported assessment design. This includes making progress visible, celebrating incremental competence gains, and linking assessment outcomes explicitly to employability contexts. For instance, vocational speaking tasks can simulate workplace dialogs, customer interactions, or interview scenarios to strengthen both expectancy (“I can perform this task”) and value (“this skill matters for my job”). Moreover, hybrid teacher-AI feedback appears most effective for sustaining motivation and perceived quality. Lo et al. (2025a,b) demonstrated that combining teacher mediation with AI analytics yielded stronger learner motivation and outcome quality than AI-only feedback. Vocational educators should thus integrate AI scoring with teacher interpretation and personalized guidance to maintain a supportive motivational climate.

Second, given that engagement (*β* = 0.319) predicted ESC but was not necessary in every high-performing configuration (OA●, MODT●, ENDT○), engagement strategies must be adaptive. For less confident learners, AI-enabled systems can provide low-pressure, repetitive practice that gradually builds self-efficacy. For more proficient learners, however, deeper engagement through peer–AI collaboration or reflective feedback cycles can enrich learning outcomes. Institutions should therefore adopt *tiered engagement models* that align engagement intensity with learners’ motivational readiness.

Third, the mediation results (H4a, H4b, and H5) demonstrate that OA exerts its influence mainly through motivational and sequential pathways rather than direct instructional effects. This calls for comprehensive *teacher training* to help educators frame AI assessments as supportive learning tools rather than punitive grading mechanisms. Without this pedagogical mediation, AI systems risk fostering compliance rather than communicative growth.

Fourth, the fsQCA configuration (~OA●MODT●ENDT●) highlights the need for *equity-focused interventions*. Since motivated and engaged learners can succeed even without extensive access to AI systems, institutions must ensure that technological disparities do not translate into learning inequities. Peer mentoring, blended practice platforms, and scaffolded motivational coaching can offer compensatory support to learners with limited technological access.

Moreover, the post-hoc analysis confirms that highly motivated and engaged learners (MODT● ENDT● OA○) achieve high ESC through self-initiated strategies such as peer conversation circles and mobile-app practice. This underscores that AI-augmented OA should be treated as an enhancer rather than a dependency: when resource constraints limit AI access, institutions can still replicate its benefits by cultivating collaborative, feedback-rich environments that sustain intrinsic motivation and engagement.

Finally, the PLSpredict results suggest that AI systems excel at predicting measurable subskills—fluency (*Q*^2^ = 0.256) and coherence (*Q*^2^ = 0.267)—but are less accurate for vocabulary and rhetorical development (*Q*^2^ ≈ 0.20). Developers should therefore prioritize *qualitative enhancements* in feedback, integrating adaptive difficulty, affective prompts, and culturally relevant examples to strengthen learners’ trust and emotional engagement.

In sum, AI-augmented OA should be viewed as part of a broader *pedagogical ecosystem* rather than a standalone innovation. Its success depends on aligning three elements: teacher mediation (motivation), institutional climate (engagement), and system design (credibility and cultural resonance). Only when these interact can AI tools transform assessment from a testing mechanism into a sustained source of communicative empowerment.

## 6 Conclusion and future research

This study examined how AI-augmented OA is associated with self-perceived English-speaking competency (ESC) among vocational EFL learners—a population often underrepresented in applied linguistics research despite their direct employability stakes. The results indicate that OA’s strongest influence lies in motivating learners and supporting engagement rather than producing direct performance gains. Motivation consistently emerged as the indispensable condition across both statistical and configurational analyses, while engagement amplified these effects but was not uniformly essential. These findings reposition OA as a *psychological catalyst* that enhances perceived competence and effort investment rather than as a purely instructional device.

The contribution of this work lies in its integrative illustration of how LOA, SDT, and engagement theory function synergistically in AI-mediated educational contexts. The evidence shows that feedback must be credible and contextually meaningful, that external incentives can constructively reinforce motivation, and that engagement operates conditionally in algorithmic environments. At a practical level, the study connects applied linguistics, vocational pedagogy, and educational technology, highlighting that the real leverage of AI assessment lies in how human, institutional, and digital components are orchestrated.

Nonetheless, several limitations warrant acknowledgment. First, ESC was measured through self-reported perceptions, which may not perfectly reflect actual communicative performance; incorporating objective speaking assessments would strengthen future analyses. Second, the cross-sectional design precludes causal inference—longitudinal and experimental research is required to track motivational and engagement dynamics over time. Third, the cultural adaptation of AI feedback tools may constrain transferability; replication in diverse linguistic and institutional contexts would test generalizability. Fourth, the study’s composite modeling of ESC should be refined by examining subskill-level effects (e.g., fluency, pragmatics, rhetorical organization) to determine whether AI assessment impacts these dimensions differently. Finally, future models should incorporate additional psychological mediators, such as language anxiety, digital literacy, and peer collaboration, to capture the fuller complexity of AI-assisted learning.

In conclusion, AI-enhanced OA represents a promising but context-sensitive mechanism for bridging skill gaps in vocational English education. Its transformative potential depends less on the sophistication of algorithms and more on the motivational, pedagogical, and institutional environments that render assessment feedback meaningful. Future research that deepens theoretical integration, expands methodological scope, and embraces cross-contextual comparison will be vital in realizing AI’s constructive role in advancing equitable and sustainable language learning outcomes.

## Data Availability

The raw data supporting the conclusions of this article will be made available by the authors, without undue reservation.
