# Supplementary material for: Motivation and engagement as pathways: how AI-augmented online assessment shapes English-speaking competency in vocational EFL classrooms
Source: Front Psychol. 2026 Jan 6;16:1730953. doi: 10.3389/fpsyg.2025.1730953 (PMC12816374; doi:10.3389/fpsyg.2025.1730953)
Supplement: Supplementary file 1 [file Supplementary_file_1.docx]

# Appendix

| **Dimension** | **Items** | **Source** |
| --- | --- | --- |
| **Online Assessment (OA) (10 items)** | | |
| **Assessment Value** | OA1. AI-based online speaking assessments improve my English-speaking proficiency.  OA2. The assessment tasks are relevant to the communication skills required in vocational and workplace settings. | Sumaharani et al. (2023) |
| **Feedback Immediacy** | OA3. I receive instant AI-generated feedback after completing speaking tasks.  OA4. The automated feedback highlights my strengths and weaknesses clearly and quickly. |  |
| **Technological Integration** | OA5. The AI-powered platform makes it easy to complete and review speaking tasks.  OA6. AI scoring and feedback are seamlessly integrated with other digital learning tools I use. |  |
| **Task-Related Motivation & Design** | OA7. AI-driven speaking tasks encourage me to practice more frequently.  OA8. Automated assessments reduce my anxiety compared to speaking in front of peers or teachers.  OA9. AI feedback increases my confidence in my speaking ability.  OA10. The adaptive design of AI tasks makes speaking practice more interactive and engaging. |  |
| **Motivation in Digital Teaching (MODT) (9 items)** | | |
| **Expectancy Beliefs / Self-Efficacy** | M1. I am confident I can perform well when speaking is assessed through AI tools.  M2. I expect to improve my oral English using AI-driven assessments.  M3. I believe I can master speaking skills evaluated by AI feedback systems. | Pintrich & de Groot (1990) |
| **Task Value / Goal Orientation** | M4. I find AI-based assessments useful for my academic and career goals.  M5. I set goals to improve my speaking performance based on AI-generated scores.  M6. I engage in AI-driven assessments not just for grades, but to develop real communication skills. |  |
| **Affective Regulation (Anxiety / Confidence)** | M7. I feel nervous when my speaking is evaluated by AI systems. (reverse)  M8. I can stay calm even when AI tasks are challenging.  M9. I am motivated to persist with AI-based assessments even when they highlight errors. |  |
| **Engagement in Digital Teaching (ENDT) (10 items)** | | |
| **Behavioral Engagement** | E1. I actively participate in AI-supported online speaking tasks.  E2. I complete AI-based speaking assessments on time.  E3. I make a strong effort to improve my AI-evaluated speaking performance.  E4. I stay focused during AI-augmented speaking sessions. | Maroco et al. (2016) |
| **Emotional Engagement** | E5. I feel excited when using AI tools for speaking practice.  E6. I enjoy learning English with AI-powered digital platforms.  E7. I feel proud when AI feedback shows my speaking has improved. |  |
| **Cognitive Engagement** | E8. I reflect on AI feedback to improve my speaking strategies.  E9. I use different strategies to handle difficult tasks identified by AI feedback.  E10. I consider how AI-evaluated tasks prepare me for real-world communication. |  |
| **English-Speaking Competency (ESC) (12 items)** | | |
| **Fluency** | S1. I can speak smoothly in English during AI-based assessments.  S2. I can maintain fluency even when under pressure in AI-monitored speaking tasks. | X. Zhang et al. (2019) |
| **Accuracy** | S3. I can use correct grammar in English when evaluated by AI.  S4. I can pronounce English words accurately in AI-monitored speaking tasks. |  |
| **Vocabulary Range** | S5. I can use appropriate vocabulary when speaking in AI-driven assessments.  S6. I can vary my vocabulary to express ideas clearly in AI-evaluated tasks. |  |
| **Coherence & Structure** | S7. I can organize my speech clearly when completing AI-assessed tasks.  S8. I can use examples and arguments effectively in AI-monitored speaking tasks. |  |
| **Rhetorical / Argument Development** | S9. I can develop ideas logically in AI-based oral assessments.  S10. I can adapt my speaking style to different AI-evaluated contexts. |  |
| **Emotional / Expressive Delivery** | S11. I can deliver my speech with appropriate tone and emotion during AI-monitored tasks.  S12. I can adjust my speaking style to maintain engagement in AI-driven assessments. |  |
